# Biomechanical assessment of disease outcome in surgical interventions for medial meniscal posterior root tears: a finite element analysis

**DOI:** 10.1186/s12891-022-06069-z

**Published:** 2022-12-14

**Authors:** Zhi Xu, Yuwan Li, Jingcheng Rao, Ying Jin, Yushun Huang, Xing Xu, Yi Liu, Shoujin Tian

**Affiliations:** 1 Department of Orthopaedics, Zhangjiagang Fifth People’s Hospital, Zhangjiagang, 215600 Jiangsu China; 2 College of Continuing Education, University of Shanghai for Science and Technology, Shanghai, 200000 China; 3 Department of Sports Medicine, Peking University Third Hospital, Institute of Sports Medicine of Peking University, Beijing Key Laboratory of Sports Injuries, Beijing, 100191 China; 4 Department of Orthopaedics, Suqian Hospital of Nanjing Drum Tower Hospital Group, Suqian, 223800 Jiangsu China; 5 Department of Orthopaedics, The Affiliated Hospital of Zunyi Medical University, No.149 Dalian Road, Zunyi, 563000 Guizhou China; 6 Department of Orthopaedics, Jen Ching memorial Hospital, Kunshan, 215300 Jiangsu China; 7 Department of Medicine, Zhijin People’s Hospital, Zhijin, 552100 Guizhou China; 8 Department of Orthopaedics, Zhangjiagang First People’s Hospital, No.68 Jiyang West Road, Zhangjiagang, 215600 Jiangsu China

**Keywords:** Posterior root tear of medial meniscus, Partial meniscectomy, meniscus repair, Finite element analysis, Biomechanics, Gait cycle

## Abstract

**Background:**

The adverse consequences of medial meniscus posterior root tears have become increasingly familiar to surgeons, and treatment strategies have become increasingly abundant. In this paper, the finite element gait analysis method was used to explore the differences in the biomechanical characteristics of the knee joint under different conditions.

**Methods:**

Based on CT computed tomography and MR images, (I) an intact knee (IK) model with bone, cartilage, meniscus and main ligaments was established. Based on this model, the posterior root of the medial meniscus was resected, and (ii) the partial tear (PT) model, (iii) the entire radial tear (ERT) model, and (iv) the entire oblique tear (EOT) model were established according to the scope and degree of resection. Then, the (v) meniscus repair (MR) model and (vi) partial meniscectomy (PM) model were developed according to the operation method. The differences in stress, displacement and contact area among different models were evaluated under ISO gait loading conditions.

**Results:**

Under gait loading, there was no significant difference in the maximum stress of the medial and lateral tibiofemoral joints among the six models. Compared with the medial tibiofemoral joint stress of the IK model, the stress of the PM model increased by 8.3%, while that of the MR model decreased by 18.9%; at the same time, the contact stress of the medial tibiofemoral joint of the ERT and EOT models increased by 17.9 and 25.3%, respectively. The displacement of the medial meniscus in the ERT and EOT models was significantly larger than that in the IK model (*P* < 0.05), and the tibial and femoral contact areas of these two models were lower than those of the IK model (*P* < 0.05).

**Conclusions:**

The integrity of the posterior root of the medial meniscus plays an important role in maintaining normal tibial-femoral joint contact mechanics. Partial meniscectomy is not beneficial for improving the tibial-thigh contact situation. Meniscal repair has a positive effect on restoring the normal biomechanical properties of the medial meniscus.

**Supplementary Information:**

The online version contains supplementary material available at 10.1186/s12891-022-06069-z.

## Introduction

Medial meniscus posterior root tears (MMPRTs) are a special type of meniscus lesion that have attracted increasing attention in the past few years. MMPRTs account for up to 20% of all meniscal tears, affecting nearly 100,000 patients each year [1]. MMPRTs lead to the destruction of annular fibres, which further makes the meniscus lose its annular tension, similar to the effect of total meniscectomy [2]. Historically, meniscectomy has been the first choice for the treatment of MMPRTs, but most cases gradually developed osteoarthritis in the few years after surgery [3, 4]. Later, as understanding of the function of the meniscus improved, partial meniscectomy became more widespread, and the load-transmitting function of a portion of the meniscus could still be preserved after surgery. However, Krych et al. [5] revealed that partial meniscectomy had no benefit in preventing the progression of osteoarthritis. In recent years, as orthopaedic surgeons have paid more attention to the integrity of the posterior root of the meniscus, meniscus repair has been given priority for MMPRT patients who meet the appropriate surgical conditions, and good results have been obtained [6].

According to a number of previous cohort and retrospective studies, partial meniscectomy is superior to meniscectomy, and meniscus repair is superior to partial meniscectomy [7, 8]. However, few studies have directly compared the results of meniscus repair and partial meniscectomy [9]. The study of the stress representation in the articular cavity can reveal the biomechanical characteristics of different surgical procedures and provide strong evidence for the interpretation of clinical results. In recent years, advances in computer simulation technology have led to the development of finite element analysis (FEA), an advanced methodology that overcomes the limitations of traditional biomechanical experiments on objective conditions and can simulate the experimental process accurately and vividly. Although the results of simulation experiments are not necessarily consistent with the facts, they can reflect the trend of stress dissimilation for different experimental subjects. We hypothesized that overloading of the tibiofemoral joint in the partial meniscectomy model was associated with partial damage to the meniscus ring with changes in joint range of motion. Elucidating this mechanical feature may help improve our understanding of the value of meniscus repair in restoring the biomechanics of the knee joint. Therefore, the main purpose of this study is to evaluate the biomechanical changes of the knee joint under different meniscus conditions in a complete gait cycle with FEA, to directly compare the difference between the two operations and to provide convincing evidence on the role of meniscus repair in restoring the physiological activity of the meniscus.

## Materials and methods

### General information

The orthopaedic clinic recruited a 25-year-old male volunteer measuring 168 cm in height and weighing 65 kg. The volunteer had no history of medical or surgical diseases and no history of knee joint injury or operation. Physical and X-ray examination ruled out acute and chronic knee joint diseases. The volunteer provided informed consent for the study and signed the informed consent form. All the methods in this study were carried out in accordance with relevant guidelines and regulations. All the experimental schemes were approved by the Institutional Review Board of Zhangjiagang Fifth People’s Hospital (L2022018).

### Acquisition of CT and MRI imaging data

The volunteer was placed in the supine position, and his right knee joint was kept relaxed and extended as it was scanned with a 1.5TMRI scanner (Siemens; Germany). The scanning range was from 83 mm above the superior edge of the patella to 92 mm below the knee joint line, covering the whole knee joint. The scanning parameters were as follows: 176 serial slices and slice thickness of 1.5 mm, repetition time 1000 ms, echo time 55 ms, acquisition matrix 240*228, pixel size 0.63 mm, and field of view 153 mm. Computed tomography was performed on the same individual using a GELightspeed16CT device (GE Healthcare, USA). The scanning parameters were as follows: layer thickness 0.9 mm, acquisition matrix ​​512 × 512, pixel size 0.705 mm, and field of view 500 mm. A total of 289 DICOM slices were obtained.

### Establishment of knee joint geometric models

The DICOM-format images of the knee joint were imported into MIMICS 19.0 software (Materialise, Belgium). The appropriate grey value was selected to distinguish bone from the surrounding soft tissue, and the instructions for the area growth and mask editing tools in the tool panel were followed to generate bone models, including models of the femur, patella, tibia and fibula. The contours of the articular cartilages and menisci were segmented from the MR images. To minimize variation in the models, manual segmentation was performed under the supervision of an experienced radiologist and orthopaedist, with an accuracy of 0.1 mm. The apparent density (ρ), Young’s modulus (E) and Poisson’s ratio of each part were calculated by the HU value of the CT scan according to the following formula [10]:

1 $$\uprho \left(\textrm{g}/{cm}^3\right)=0.000968\ast \textrm{HU}+0.5$$

2 $$\textrm{If}\ \rho <1.2\textrm{g}/{cm}^3,\textrm{E}=2014{\rho}^{2.5}\left(\textrm{MPa}\right),\textrm{v}=0.2$$

3 $$\textrm{If}\ \rho >1.2\textrm{g}/{cm}^3,\textrm{E}=1793{\rho}^{3.2}\left(\textrm{MPa}\right),\textrm{v}=0.32$$

**Fig. 1:**
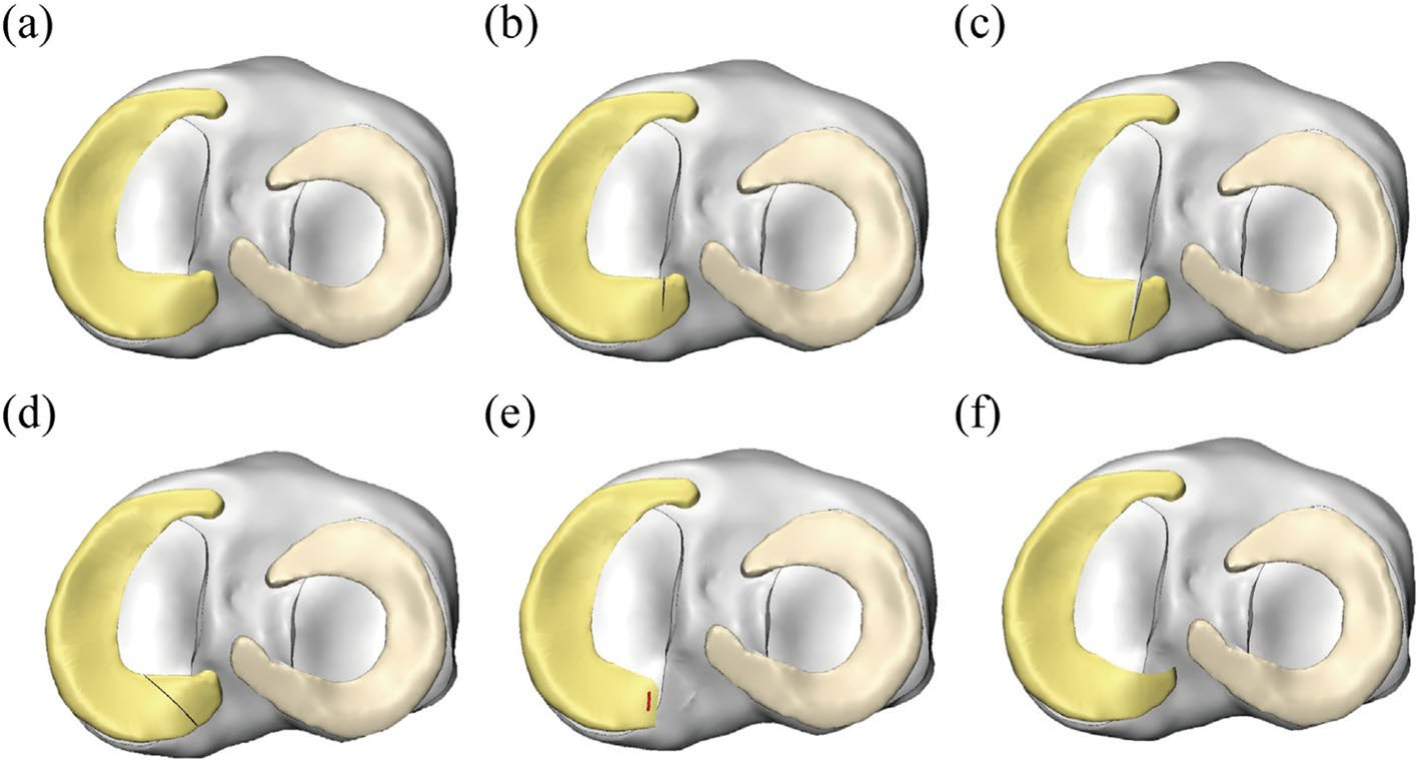
Three dimensional reconstructed models of the knee joint used in the analysis for the **a** IK intact knee model; **b** PT partial tear model; **c** ERT entire radial tear model; **d** EOT entire oblique tear model; **e** MR meniscus repair model; **f** PM partial meniscectomy model

Then, each segmented 3D assembly was saved in STL format, and the STL file was imported into GeomagicWrap2017 software (Geomagic company, USA). NURBS surface files of the bones were acquired by processing the images via unification, removal of external solitary points, noise reduction, and packaging and surface fitting. The surface patches were rasterized, and a complete NUBERS surface file was formed and saved as 6 models. The above files were then opened in turn in Pro/E5.0 software (PTC company, USA), and then the medial meniscus was cut and assembled according to the content of the experiment. Six solid models of knee joints were established, and each part was saved in IGS format. The knee joint components were imported into 3-Magic software (Materialise company, Belgium) for assembly display. (i) An intact knee (IK) model was developed (Fig. 1a). To construct the pathological meniscus posterior root tear model, we referred to the research results of Dr. Laprade RF’s group [11], who divided lesions into five types according to the shape and location of the medial meniscus posterior root tear: type 1 (7.0% of all root tears): partial stable meniscal tear 0 to 9 mm from the root attachment; type 2 (67.6% of all): complete radial meniscal tear; type 3 (5.6% of all): bucket-handle tear with meniscal root detachment; type 4 (9.9% of all): complex oblique meniscal tear extending into the root attachment; type 5 (9.9% of all): avulsion fracture of the meniscal root attachment. We believe that the incidence of type 3 lesions is low, and the mechanism of lesions is more complex, so it is not discussed in this study. In addition, the effect of type 5 is similar to that of type 2-3 (i.e., the posterior root completely loses fixation), and thus a separate model does not need to be established. According to the actual needs of this study, three pathological models were established: (ii) the partial tear (PT) model (Fig. 1b), (iii) the entire radial tear (ERT) model (Fig. 1c), and (iv) the entire oblique tear (EOT) model (Fig. 1d). Given that type 2 lesions are the most common, using the ERT model, we removed the white zone of the stump of the posterior root to “freshen” the local tissue and then sutured and riveted the remaining tissue to establish (v) the meniscus repair (MR) model (Fig. 1e). Using the PT model, the soft tissue in the injured area was partially excised to smooth the surface of the posterior root of the meniscus to develop (vi) the partial meniscectomy (PM) model (Fig. 1f).

### FE modelling and material properties

**Fig. 2:**
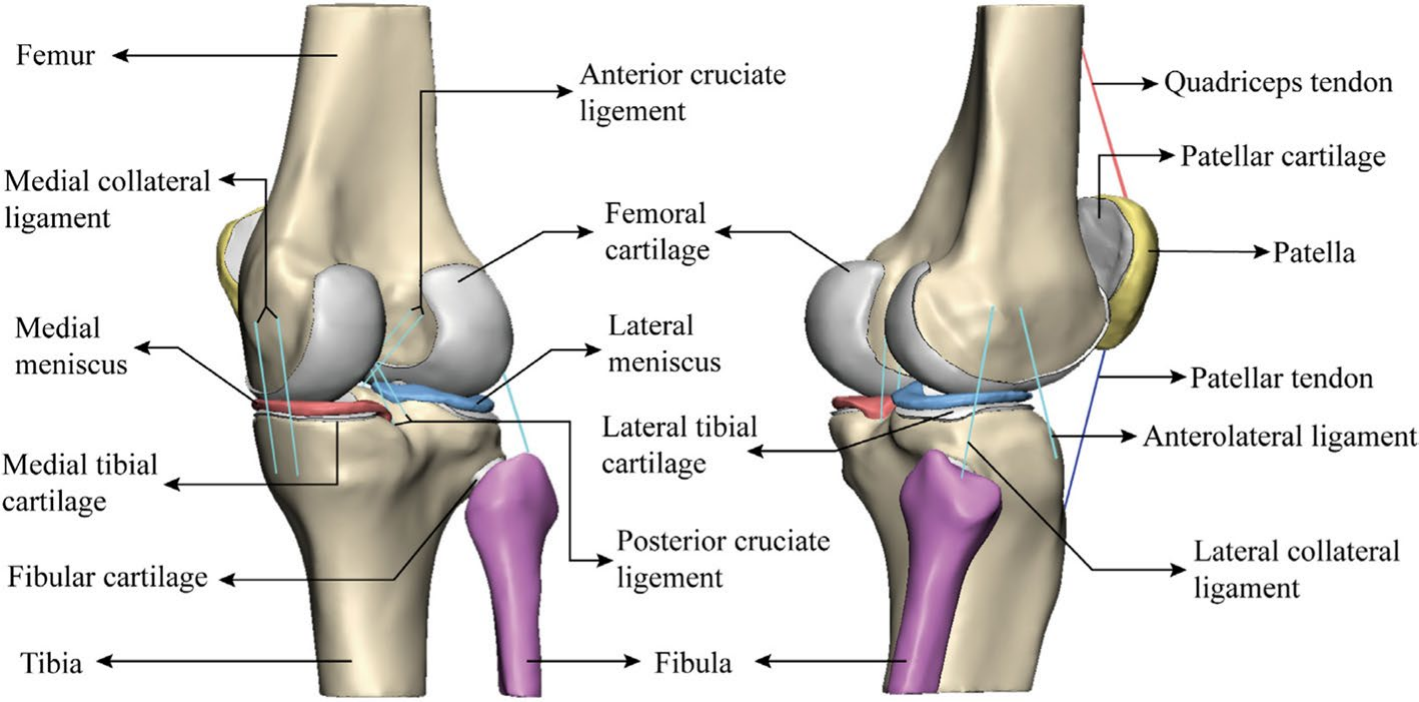
Anatomical structure display of three-dimensional reconstruction of the knee joint

The solid parts of the CAD knee joint were imported into the HyperMesh2014 software (Altair company, USA) in IGS format, and the four-node linear tetrahedron (C3D4) fully integrated element was selected for meshing. To optimize the grid density for ensuring calculation accuracy, we first sought to verify the grid sensitivity of the model and set the knee joint model under a 1000 N axial compression load. The error of the peak stress of the medial tibiofemoral joint was within 5%, which is acceptable for establishing multiple grid models from coarse to fine. The mesh size of the bony structure was 2 mm, and the mesh size of the soft tissue structure was 0.8 mm. The divided mesh parts were saved as INP files and imported into Abaqus 6.14 (Dassault company, France) in the finite element analysis software for material assignment. Considering that the ligament tissue can only withstand tension but not compression, early studies have shown that the ligament tissue is a nonlinear material, and thus nonlinear elastic properties with slack regions were generated by defining the force-extension relationship of the ligament [12]:

4 $$f\left(\upvarepsilon \right)\left\{\begin{array}{cc}\frac{k{\upvarepsilon}^2}{4{\upvarepsilon}_1},& 0\le \upvarepsilon \le 2{\upvarepsilon}_1\\ {}k\left(\upvarepsilon -{\upvarepsilon}_1\right),& \upvarepsilon >2{\upvarepsilon}_1\\ {}0,& \upvarepsilon <0\end{array}\right.$$

where *f* is the current force, *k* is the spring stiffness, *ɛ* is the strain, and *ɛ*_*1*_ is the nonlinear strain parameter. The relevant parameters were obtained by Blankevoort et al. Each nonlinear spring was connected to a suitable location on the finite element model under the guidance of clinicians and radiologists (Fig. 2).

Articular cartilages were modelled as neo-Hookean hyperelastic isotropic material (nonlinear) with the strain energy density as a function of elastic volume strain (*J*_*el*_) and first strain invariant (${\overline{I}}_1$) [13]:

5 $$U={C}_{10}\left({\overline{I}}_1-3\right)+\frac{1}{D_1}{\left({J}_{el}-1\right)}^2$$

In eq. (5), *C*_10_ and *D*_1_ are neo-Hookean material constants, which are reported in the references (*C*_10_ = 0.86 MPa and *D*_1_ = 0.048 MPa^− 1^).

By combining the strain energy density function with the Holzapfel-Gasser-Ogden (HGO) material model, transversely isotropic hyperelastic meniscal materials can be constructed [14]:

6 $$U={C}_{10}\left({\overline{I}}_1-3\right)+\frac{1}{D_1}\left(\frac{{\left({J}_{el}\right)}^2-1}{2}-\textrm{In}\ {J}_{el}\right)+\frac{k_1}{2{k}_2}\left(\exp \left[{k}_2{\left\langle {\overline{E}}_{\alpha}\right\rangle}^2\right]-1\right)$$

with

7 $${\overline{E}}_{\alpha }=\kappa \left({\overline{I}}_1-3\right)+\left(1-3\kappa \right)\left({\overline{I}}_{4\left(\alpha \alpha \right)}-1\right)$$

In eq. (7), ${\overline{I}}_{4\left(\alpha \alpha \right)}$ is a pseudoinvariant of the symmetrically modified Cauchy-Green strain tensor, which simulates hard elastic collagen fibres. Among the parameters, *C*_10_, *D*_1_, *k*_1_, *k*_2_ and *κ* (Table 1) are used by Abaqus software to simulate real hyperelastic material properties in the calculation. In this study, meniscal fibres were circumferentially aligned (*κ* = 0) to resist circumferential stress during cyclic loading of the gait. The material properties of bone and sutures were determined based on previously published data and were defined as isotropic linear elastic materials with the parameters shown in Table 2.

**Table 1:** Material parameters used for modeling the medial and lateral meniscus. Parameters *C*_10_ and *D*_1_: neo-Hookean constants, *k*_1_ and *k*_2_: HGO coefficients, and *κ*: Fiber dispersion and orientation level [15, 16]

Components	*C*_10_ (MPa)	*D*_1_ (MPa^− 1^)	*k*_1_	*k*_2_	*κ*
Medial meniscus	1	5e-3	5.0	0.9	0
Lateral meniscus	1	5e-3	8.5	1.6	0

**Table 2:** Material properties

Components	Elastic modulus (MPa)	Poisson’s ratio	Element	Reference
Femur	17,000	0.30	21,965	[17]
Tibia	14,000	0.30	20,011	[17]
Fibula	11,000	0.30	9451	[18]
Patella	11,000	0.30	9235	[18]
Suture	380,000	0.39	1774	[19]

### Loads and boundary conditions

**Fig. 3:**
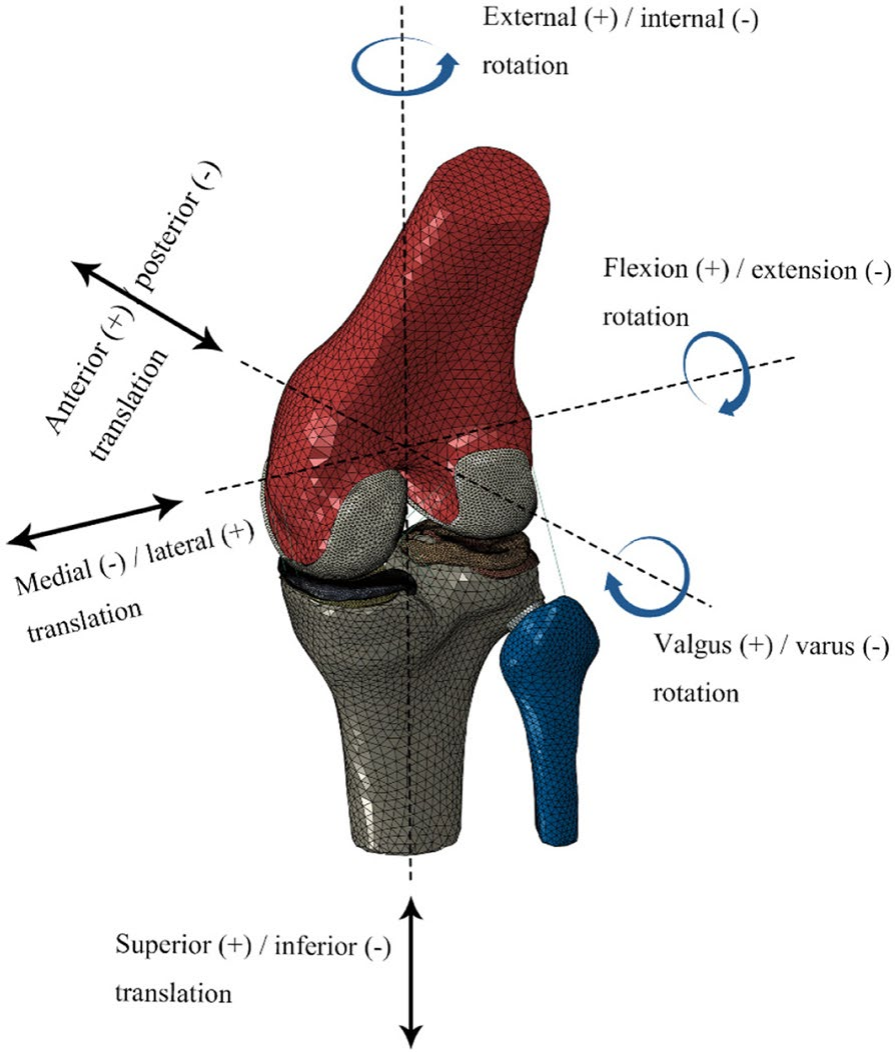
finite element mesh model of the knee joint

**Fig. 4:**
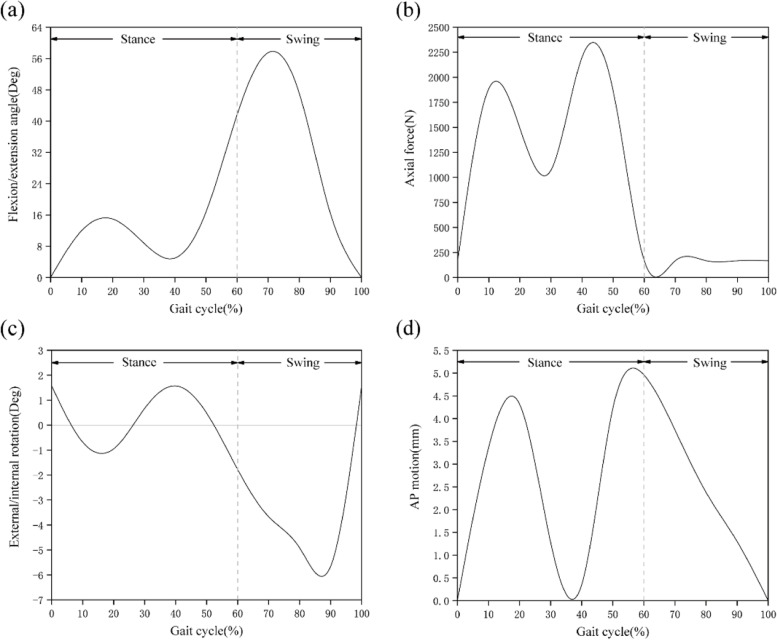
Input function for finite element model based on the ISO gait cycle: **a** Flexion angle; **b** Axial load; **c** Internal–external rotation; **d** Anterior–posterior displacement. Reprinted with permission from ISO, ISO 14243-3: 2014. Copyright (2014) International Organization Environmental Standardization

The assembly of the knee joint finite element model was completed in the assembly module of the Abaqus 6.14 software, as shown in Fig. 3. The pathological model was developed by replacing the normal meniscus with the medial meniscus injury model based on the intact model. After the assembly was completed, the Interaction module was loaded and 6 groups of contact surfaces were set: the medial compartment, including femoral cartilage - tibial cartilage, femoral cartilage - medial meniscus, and medial meniscus - tibial articular cartilage surfaces; and the lateral compartment, including femoral cartilage - tibial cartilage, lateral meniscus - femoral cartilage, and lateral meniscus - tibial cartilage surfaces. A joint interface was defined as a hard contact with *μ* = 0.002 friction coefficient [20], no penetration, and limited slip. The bottom ends of the tibia and fibula were fully fixed in 6 degrees of freedom. To make the simulation results closer to reality, the gait load and displacement were set according to ISO 14243-3:2014 for the development of knee joint prostheses to simulate the activities of the knee joint in the complete gait cycle, which is helpful for studying the biomechanical characteristics of the knee joint in various phases. The ISO standard gait parameter curve is shown in Fig. 4.

### Model validation

To verify the effectiveness of our finite element model of the knee joint, in this study, the reported stress results of the femoral cartilage, meniscus and tibial cartilage measured in knee flexion at 0 degree posture were compared with previous studies [21, 22], and the results were found to be similar (Table 3). We measured the peak stress of the medial/lateral compartments of the knee joint model as 7.9 MPa/6.3 MPa; given that the peak range of contact pressure of the medial/lateral compartment measured in biomechanical experiments on cadaveric specimens was 6-11 MPa/5-10.5 MPa [23], the contact stress of this model was within a reasonable range. In addition, in the numerical verification of the area, the total contact area of the medial/lateral intercompartment measured was 737.6 mm^2^ / 550.6 mm^2^, which is consistent with a previous report [24, 25] that found a total contact area of 650 ± 190 mm^2^/500 ± 90 mm^2^, respectively. The contact area of the medial compartment was higher than that of the lateral compartment. After a comparison with previous studies, we considered the knee joint finite element model we developed to be suitable and sufficiently robust for further research.

**Table 3:** Validation of stress (MPa) in finite element models

Studies	SSFC	SSM	SSTC	CSFC	CSM	CSTC
Our model	1.72	11.45	3.56	6.51	6.61	8.45
Zhang K et al	2.00	6.72	2.40	4.25	9.15	6.81
Shriram D et al	1.93	–	2.32	2.76	–	3.52

*SSFC* maximum shear stress on femoral cartilages, *SSM* maximum shear stress on meniscus, *SSTC* maximum shear stress on tibial cartilages, *CSFC* maximum compressive stress on femoral cartilages, *CSM* maximum compressive stress on meniscus, *CSTC* maximum compressive stress on tibial cartilages

## Results

### Comparison of contact mechanics

**Fig. 5:**
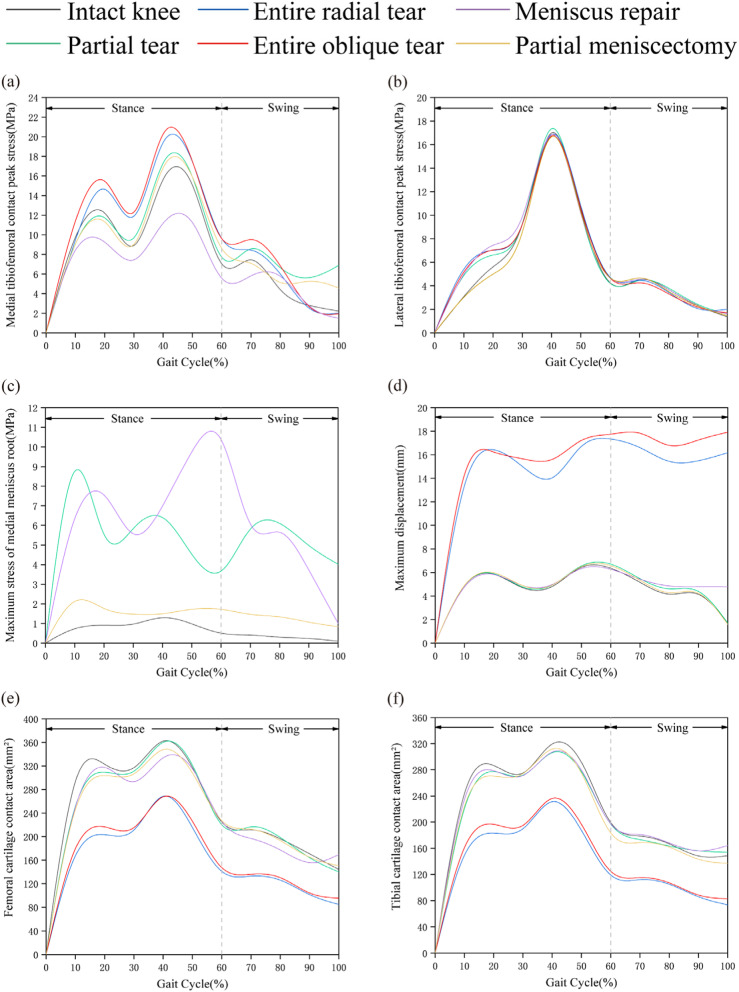
Biomechanical properties comparison under the gait period in finite element models. **a** Medial tibiofemoral contact peak stress; **b** Lateral tibiofemoral contact peak stress; **c** Maximum stress of medial meniscus root; **d** Maximum displacement on medial meniscus; **e** Contact area of femoral medial cartilage; **f** Contact area of tibial medial cartilage

**Fig. 6:**
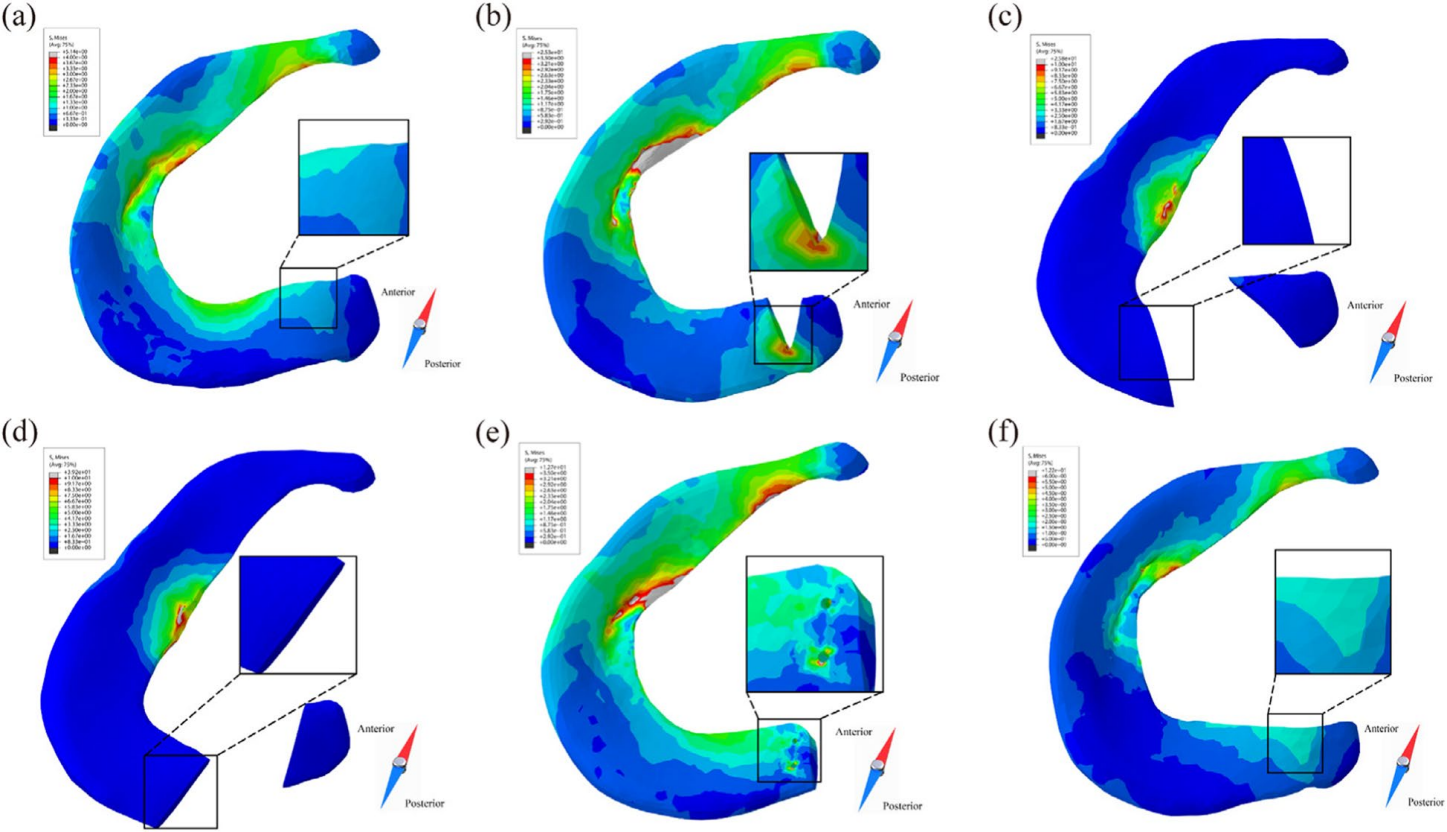
stress distribution of posterior root of medial meniscus in different models at 10% of the gait cycle. **a** IK intact knee model; **b** PT partial tear model; **c** EOT entire oblique tear model; **d** ERT entire radial tear model; **e** MR meniscus repair model; **f** PM partial meniscectomy model

**Fig. 7:**
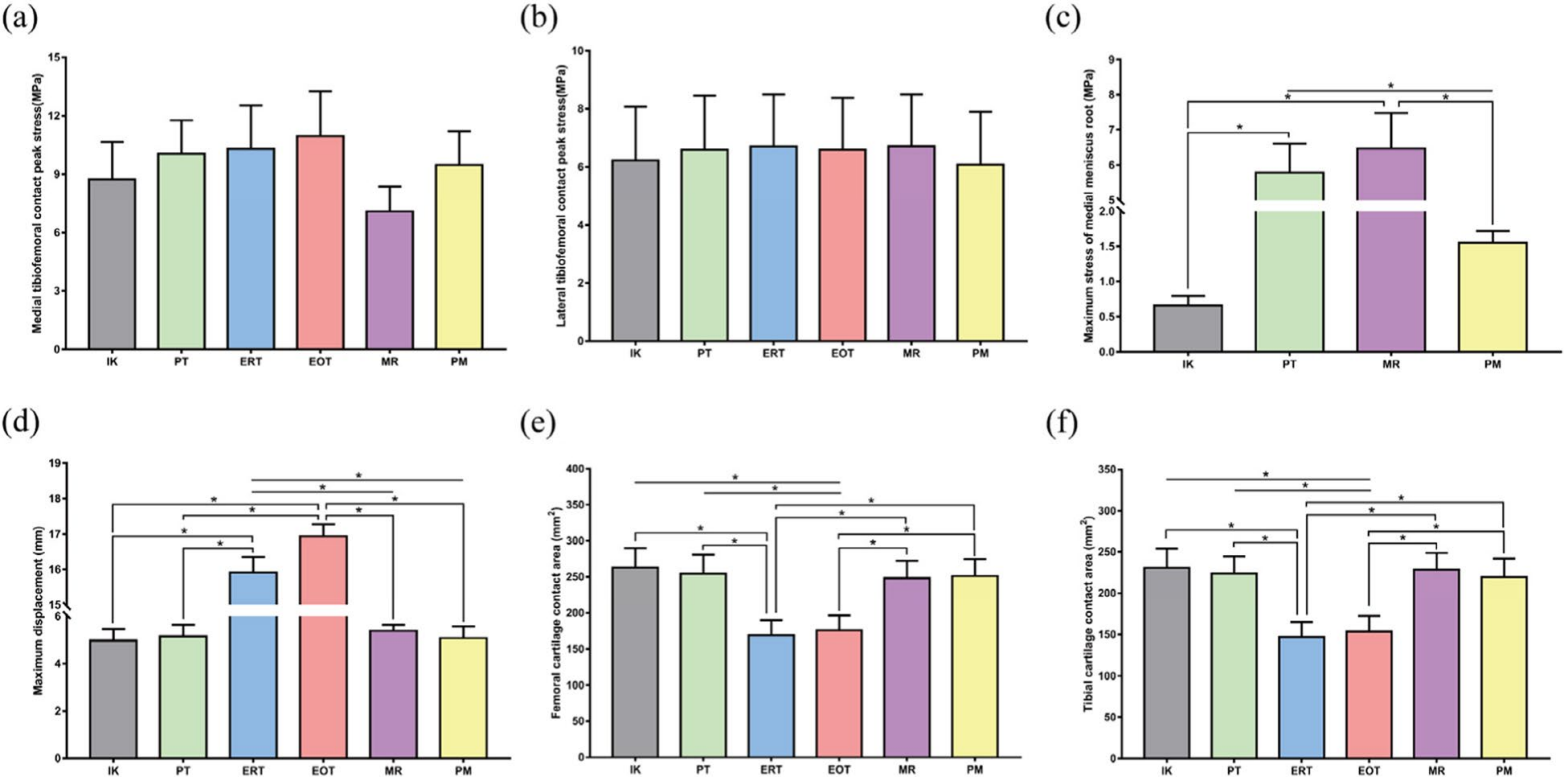
Stress, displacement and contact area analysis in different models. IK intact knee, PT partial tear, ERT entire radial tear, EOT entire oblique tear, MR meniscus repair, PM partial menisectomy. **a**, **b** There was no significant difference in the contact stress between the medial and lateral tibiofemoral articular cartilage surfaces in different models. **c** The medial meniscus posterior root stress in the PT and MR models was higher than that in the IK and PM models. **d** The displacement of the medial meniscus in the ERT and EOT models was greater than that in the IK, PT, MR, and PM models. **e**, **f** The femoral and tibial cartilage contact areas of the ERT and EOT models were smaller than those of the other four models. *P<0.05

During the complete gait cycle, the contact stress of the 6 models was evaluated and analysed. The contact stress curves of the medial tibiofemoral joint of the six models have similar fluctuations, reaching the first and second peaks during the support phase approximately 20 and 40% of the time, respectively (Fig. 5a). Compared with that of the IK model, the contact stress of the ERT and EOT models increased by 17.9 and 25.3%, respectively, while that of the PM model increased by 8.3% and that of the MR model decreased by 18.9% (Fig. 7a). The joint contact stress of all the models reached the stress peak at 40% of the support phase (Fig. 5b), and there was no statistically significant difference in stress among the models (Fig. 7b). We measured the local maximum stress of the medial dorsal root of the medial meniscus in the IK, PT, MR and PM models. Figure 5 shows that the stress of the medial posterior root of the medial meniscus in the IK model increased in the support phase and decreased in the swing phase. It can be observed from the stress nephogram that the medial meniscus body and inner ring fibres provided the main contribution to the resistance to femoral cartilage extrusion (Fig. 6a). The local stress in the posterior root of the PT model was consistently high during the gait cycle. The stress cloud map shows the stress concentration at the edge of the posterior root fissure (Fig. 6b); compared with that of the IK model, the stress value increased by 775.8% (Fig. 7c). The posterior root stress of the MR model peaked at the end of the support phase and then decreased in the swing phase. Because the hardness of the suture material was higher than that of the surrounding soft tissue, the stress became concentrated around the suture hole (Fig. 6e), and the stress value was higher than that of the IK model by 880.3% (Fig. 7c). Compared with the IK model, the PM model was less sensitive to posterior root contact mechanics, showing only slight changes (Fig. 5c). In addition, the stress distribution in the inner meniscus of the IK and MR models was uniform, while the stress in the medial meniscus of the PM model shifted to the anterior and posterior horns (Fig. 6f).

### Kinematic comparison of the medial meniscus

Figure 5d shows that the variation in the medial meniscus displacement of the IK, PT, MR and PM models is similar under the gait cycle, while the ERT and EOT models have a large increase in the medial meniscus displacement due to root fracture. The posterior root of the medial meniscus was extended due to compression of the articular cartilage (Fig. 6c-d). Compared with that of the IK model, the displacement of the medial meniscus in the ERT and EOT models increased by 220.1 and 240.8%, respectively (Fig. 7d).

### Comparison of medial tibiofemoral articular cartilage contact area

By observing the changes in the contact area of ​​the medial femoral cartilage in the different models during the gait cycle, the contact area of ​​the six models increased with increasing axial stress during the support phase, peaked at approximately 40% of the phase, and then gradually decreased (Fig. 5e). Compared with that of the IK model, the femoral cartilage contact area of ​​the PT, MR and PM models was not significantly different. It is possible that the femoral cartilage contact area is not closely related to local injury and surgical factors, while the femoral cartilage contact area of ​​the ERT and EOT models is greatly reduced. With respect to the IK model, the reductions were 35.7 and 33.2%, respectively (Fig. 7e). Analysis of the changes in the medial tibial cartilage contact area in the six models also yielded a change pattern similar to the former (Fig. 5f). Compared with that of the IK model in this study, the tibial cartilage contact area was reduced by 36.3 and 33.4% in the ERT and EOT models, respectively (Fig. 7f).

## Discussion

The main finding of this study is that the MMPR can still generate annular tension after a partial tear or partial meniscectomy and maintain tibiofemoral joint consistency, which indicates that the difference is not obvious compared with a healthy meniscus in terms of contact mechanics, kinematics and contact area. The two entire MMPR tear models showed increased maximum stress and displacement of the tibiofemoral joint and decreased the cartilage contact area with respect to the intact model. Meniscal repair can effectively restore the mechanical properties of the meniscus.

The association of medial meniscal root tears with knee osteoarthritis (OA) progression has been well demonstrated in clinical studies [26]. Lesions that lead to tears at the root of the meniscus often occur during squats or activities involving flexion, often concurrently with some type of rotation [27]. The circumferential fibres of the meniscus disperse the vertical compressive force evenly around it, effectively improving the axial stress overload [28]. Dispersal of this axial load is essential for the viability and function of articular cartilage [29]. Root tears result in destruction of the meniscus annular structure, increased contact pressure on the tibiofemoral articular cartilage, and accelerated joint degeneration [30]. To observe this phenomenon, our team vividly simulated the changes in the biomechanical properties of the knee joint before and after an MMPR tear by constructing a gait analysis simulation model of the intact knee joint and more intuitively displayed the circumference of the meniscus to observe the effects of fibre damage on knee biomechanics. The Mises cloud map shows that after the injury, the local stress of the MMPR increases significantly around the base of the crack so that a partial tear of the meniscus can further develop into a complete tear (Fig. 6b).

Historically, partial meniscectomy for root tears has usually provided short-term relief. Krych et al. [5] followed up 52 patients with MMPRTs who underwent partial meniscectomy for 2.3-9.3 years, with an average IK DC score of 67.8. Lee et al. [31] treated 288 patients, and the overall improved Lysholm score increased from 64.4 to 81.3. However, arthroscopic partial meniscectomy for irreparable meniscal tears only offers pain relief operation and cannot stop the progression of arthritis [32, 33], and because this procedure does not fully restore meniscal ring tension, in most cases, it will eventually develop into degenerative osteoarthritis [34]. To further understand the role of partial meniscectomy in the gait movement of the knee joint, the stress in the medial compartment of the partial meniscectomy model was decreased compared with that of the entire tear model. This improvement is related to the fact that the operation retains the continuity of the root tissue, and the residual meniscus ring still plays a braking role in the process of gait, which is of positive significance for the prevention of meniscus extrusion. It is worth noting that in the measurement of the maximum stress of the tibial-femoral joint, the stress of the PM model was slightly higher than that of the IK model in the support phase and almost twice that of the IK model at the end of the swing phase (Fig. 5a). Considering that partial meniscectomy is not designed to restore biomechanics and that there is a high conversion rate to total knee arthroplasty (TKA) [5], the repair strategy is selected in the treatment of MMPRT patients. It is very significant to restore the integrity of the posterior root of the meniscus.

Chung et al. [8] followed up MMPRT patients who underwent partial meniscectomy and meniscus repair for 10 years. The Lysholm and IKDC scores at the last follow-up in the meniscus repair group were significantly higher than those in the partial meniscectomy group, and 56% of the patients in the latter group received total knee arthroplasty, compared with 22% in the former group. The results show that root repair is better than partial meniscectomy. From a long-term perspective, it is more valuable to repair the annular structure of the meniscus. Previous biomechanical studies have shown that repairing the MMPR can restore the ability to absorb circumferential stress and reduce the contact pressure of the tibiofemoral joint, equivalent to that of the natural knee joint [35]. To observe the phenomenon of meniscus repair to restore joint contact behaviour, we focused on the stress difference between the MR model and IK model and found that the maximum tibiofemoral stress of the MR model was almost lower than that of the IK model (Fig. 5a) during the gait cycle. The consequence of this finding is that part of the stress of the tibiofemoral articular surface is transferred to the posterior root when the overall load is constant. It can be clearly observed that stress concentration (Fig. 6e) occurs around the suture hole, which can be attributed to the use of rivet sutures instead of normal meniscus posterior root tissue. The stiff suture material and the concentration of fixed points are mainly responsible for the increase in local stress in the posterior root. It is gratifying to note that after meniscus repair, the overall displacement and contact area of the model almost returned to normal, consistent with previous reports [35].

A recent cohort study by Bernard et al. [36] divided 45 patients with MMPRTs into groups. At the last follow-up, patients in each group received TKA: the nonoperative group (*n* = 4), partial meniscectomy group (*n* = 9) and meniscus repair group (*n* = 0). Chung et al. [37] used survival analysis to compare the difference in TKA conversion rate between partial meniscectomy and meniscus repair. The results showed that the overall Kaplan–Meier survival probability after partial meniscectomy was 90% at 3 years, 80% at 4 years, 75% at 5 years, and 67.5% at 6 years, while that after meniscus repair was 100% by at least 5 years (*P* < 0.001). The data reflect the advantages of meniscus repair in slowing the progression of arthritis. Combined with the results of our computer simulation, although the overall performance of the PM model was similar to that of the IK model and was negative only in the later stage of the wobble phase, the factors that affect the progression of OA are diversified, and the accumulation and amplification of adverse factors are unfavourable to the health of MMPRT patients. Compared with the limited effect of partial meniscectomy in relieving pain, meniscus repair can restore the biomechanical properties of the meniscus and is of greater value in preventing OA.

This study has some limitations. First, the study utilized the imaging data provided by only one volunteer to establish a three-dimensional knee joint model. The evolution of the disease is affected by many factors, such as the baseline data of the patient, the proficiency of the surgeons, and postoperative rehabilitation management. Whether this conclusion is applicable to explain the efficacy of surgical intervention in patients with different MMPRTs needs to be further observed and confirmed. Second, at present, there are abundant technical means to restore the integrity of the posterior root of the meniscus, including rivet suture fixation technology, pull-out suture fixation technology, meniscus transplantation technology and so on. In this study, rivet suture fixation technology was selected for the meniscus repair model, so the conclusion cannot completely cover the scope of potential operations. Third, the validity verification of the finite element model in this study only refers to the research data of others and does not attempt to verify the in vitro biomechanics, considering that the factors affecting the experiment are diverse, which may affect the accuracy of the experimental results. In view of the limitations of the research design, the author’s research team plans to coordinate computer simulation and biomechanical experiments of cadaveric specimens in the future to test several groups of knee joint samples and to observe the knee biomechanical changes of each group in different activity scenes to facilitate the scientific evaluation of different surgical methods. In addition, we will perform prospective and retrospective studies on different surgical methods to delay the progression of osteoarthritis, which can further verify the conclusions of this study.

## Conclusion

The integrity of the MMPR plays a pivotal role in maintaining normal tibiofemoral joint contact stress, area and positional relationship. Once its integrity is completely or partially destroyed, the load on the medial tibiofemoral articular cartilage surface will increase. Given that partial meniscectomy is not beneficial for improving tibiofemoral contact, meniscus repair can restore the ability of the meniscus to absorb annular stress and effectively reduce the contact pressure of the tibiofemoral joint.

## Supplementary Information

**Additional file 1.**

## Data Availability

The datasets used and/or analysed during the current study are available from the corresponding author upon reasonable request.

## Abbreviations

MMPRTs: Medial meniscus posterior root tears
FEA: Finite element analysis
DICOM: Digital Imaging and Communications in Medicine
CT: Computed tomography
ISO: International Organization for Standardization
PT: Patellar tendon
QT: Quadriceps tendon
ACL: Anterior cruciate ligament
PCL: Posterior cruciate ligament
MCL: Medial collateral ligament
LCL: Lateral collateral ligament
