# Supplementary material for: Biomechanical assessment of disease outcome in surgical interventions for medial meniscal posterior root tears: a finite element analysis
Source: BMC Musculoskelet Disord. 2022 Dec 14;23:1093. doi: 10.1186/s12891-022-06069-z (PMC9749342; doi:10.1186/s12891-022-06069-z)
Supplement: Supplementary file 1 — Additional file 1. [file 12891_2022_6069_MOESM1_ESM.zip › Submission graphs and supplementary material/Supplementary material/Legends.docx]

**Legends**

“Mesh division of knee joint components” **description:** According to the request of the reviewer “l.138 Please include such detail information in the supplementary material**”,** this document supplements the node and element information of each part of the finite element model.

“Original data” **description:** The raw data used by figure5 and figure7 in this article.

“Tibia and tibial cartilage restraint test data” **description:** This document is based on the request of the reviewer**“**Your meniscectomy model indicated a lower peak stress increase compared to the repair and tear models. Clarify.l.215 Your peak stress levels at the medial and lateral compartment are manyfold higher compared to in-vitro test results. How can you explain this?**”**To explore the difference between the simulation data of this study and the experimental data in vitro, it is found that under the condition of ignoring the tangential stress of the joint surface and measuring only the axial compressive stress, the simulation data are similar to those of the previous study. it is inferred that the pressure sensor in vitro can only measure the axial positive pressure in the joint. And the result data of the simulation test are reported.
