# Supplementary material for: Biomechanical assessment of disease outcome in surgical interventions for medial meniscal posterior root tears: a finite element analysis
Source: BMC Musculoskelet Disord. 2022 Dec 14;23:1093. doi: 10.1186/s12891-022-06069-z (PMC9749342; doi:10.1186/s12891-022-06069-z)
Supplement: Supplementary file 1 — Additional file 1. [file 12891_2022_6069_MOESM1_ESM.zip › Submission graphs and supplementary material/Supplementary material/Mesh division of knee joint components.docx]

Mesh division of knee joint components

| Component | Element | Node | Grid size |
| --- | --- | --- | --- |
| Femur | 21965 | 4991 | Bone structure2mm |
| Tibia | 20011 | 4535 |  |
| Patella | 9235 | 2161 |  |
| Fibula | 9451 | 2265 |  |
| Femoral cartilage | 31558 | 10942 | Soft tissue str0.8mm |
| Tibial cartilage | 21052 | 7069 |  |
| Menisci | 67175 | 16805 |  |
